# Flow cytometric monitoring of influenza A virus infection in MDCK cells during vaccine production

**DOI:** 10.1186/1472-6750-8-45

**Published:** 2008-04-30

**Authors:** Josef Schulze-Horsel, Yvonne Genzel, Udo Reichl

**Affiliations:** 1 Bioprocess Engineering, Max Planck Institute for Dynamics of Complex Technical Systems, Sandtorstrasse 1, 39106 Magdeburg, Germany; 2 Lehrstuhl fuer Bioprozesstechnik, Otto-von-Guericke-Universitaet Magdeburg, P.O. box 4120, 39016 Magdeburg, Germany

## Abstract

**Background:**

In cell culture-based influenza vaccine production the monitoring of virus titres and cell physiology during infection is of great importance for process characterisation and optimisation. While conventional virus quantification methods give only virus titres in the culture broth, data obtained by fluorescence labelling of intracellular virus proteins provide additional information on infection dynamics. Flow cytometry represents a valuable tool to investigate the influences of cultivation conditions and process variations on virus replication and virus yields.

**Results:**

In this study, fluorescein-labelled monoclonal antibodies against influenza A virus matrix protein 1 and nucleoprotein were used for monitoring the infection status of adherent Madin-Darby canine kidney cells from bioreactor samples. Monoclonal antibody binding was shown for influenza A virus strains of different subtypes (H1N1, H1N2, H3N8) and host specificity (human, equine, swine). At high multiplicity of infection in a bioreactor, the onset of viral protein accumulation in adherent cells on microcarriers was detected at about 2 to 4 h post infection by flow cytometry. In contrast, a significant increase in titre by hemagglutination assay was detected at the earliest 4 to 6 h post infection.

**Conclusion:**

It is shown that flow cytometry is a sensitive and robust method for the monitoring of viral infection in fixed cells from bioreactor samples. Therefore, it is a valuable addition to other detection methods of influenza virus infection such as immunotitration and RNA hybridisation. Thousands of individual cells are measured per sample. Thus, the presented method is believed to be quite independent of the concentration of infected cells (multiplicity of infection and total cell concentration) in bioreactors. This allows to perform detailed studies on factors relevant for optimization of virus yields in cell cultures. The method could also be used for process characterisation and investigations concerning reproducibility in vaccine manufacturing.

## Background

Today, human influenza vaccines are still mainly produced in embryonated hen's eggs. This production system has certain disadvantages. The amount of vaccine produced is limited to the availability of embryonated eggs, which might be a problem in case of increased demand for vaccination, e.g. during a pandemic [1,2]. Furthermore, the egg-based passage of virus can lead to altered hemagglutinin compared to the original wild-type virus, which can have an impact on immunogenicity of the produced vaccines [3]. Currently, strong efforts are put into the development of cell culture-based vaccine production systems to overcome such limitations and drawbacks [1]. Several cell lines have been characterised for industrial influenza virus production, such as Vero, the human foetal retina cell line PER.C6 and Madin-Darby canine kidney (MDCK) cells [4-7].

Additionally to biochemical engineering approaches, investigation of cellular processes during viral infection is of great importance for process optimisation. For this purpose, monitoring of influenza virus production and spread of the infection on a cellular level could provide essential information. Furthermore, qualitative and quantitative monitoring of influenza virus infections is of interest for *in vitro *studies in virological and medical research.

Monitoring of influenza virus production and spread of the infection can also be useful for established vaccine production processes. There, it might be used to characterize variations in between process batches with regard to reproducibility and standardisation as recommended in the process analytical technology (PAT) guidelines by the Food and Drug Administration (FDA) [8].

Numerous methods for the assessment of influenza A virus infection in *vitro *have been established over the years. Widespread classical methods are based on titrations of virus particles in tissue-culture supernatant [9,10].

The hemagglutination assay quantifies the concentration of infectious and non-infectious virions via binding to erythrocytes [10,11]. In influenza virus diagnosis and quantification in clinical samples and cell culture supernatants, quantitative real-time PCR is widely used [12-16]. Other, more sophisticated methods for the determination of total virus titres implement single nanometric particle enumerators [17] or microsphere-based flow cytometric immunoassays [18]. The concentration of infectious virus particles is commonly determined either with a plaque assay [19,20], or as tissue-culture infectious dose (TCID_50_) [10]. Titrations of virus particles in tissue-culture supernatant depend on release of virus particles from infected cells, which is a late event in the course of influenza virus infection. The preceding stages during influenza virus infection are virus genome replication, transcription and translation [21,22]. The detection of viral RNA extracted from tissue-cultures, using RNA hybridisation, is a method for the detection of influenza virus replication [23].

The translation of viral mRNA can be detected via immunofluorescence microscopy of virus proteins. This can be done either using polyclonal [24] or monoclonal antibodies [23,25]. A comparison of the hemagglutination assay with RNA hybridisation, titration of infectious virus and immunofluorescence microscopy using a fluorochrome-labelled monoclonal antibody was described by Rimmelzwaan et al. [23]. RNA hybridisation, titration of infectious virus and immunofluorescence microscopy showed equal sensitivity, exceeding the sensitivity of the HA assay.

The monitoring and quantification of host-cell infection during cell culture-based influenza A virus production needs to meet several goals: Preferably, it should be sensitive, quantitative and robust enough to handle bioprocess modifications such as differences in multiplicity of infection (moi) or cell concentration at time of infection. The assay should be applicable to influenza A virus strains of different host species to cover human and veterinary influenza vaccine manufacturing. Furthermore, the assay should allow monitoring of different virus subtypes to comply with the annual recommendations of the World Health Organization for human vaccines [2]. Finally, the assay should be of use for monitoring bioprocesses with respect to the PAT guidelines [8]. In general, bioreactor samples are taken at different time-points during the infection and should be measured simultaneously to reduce preparation effort and to increase comparability within samples. It is therefore required that the assay allows the measurement of fixed cells. Additionally, the staining procedure should be as rapid as possible and allow co-staining against other physiological parameters. For biosafety reasons and to facilitate sample handling, virus inactivation prior to sample preparation would be preferable. Compared to fluorescence microscopy, flow cytometry allows acquisition of statistically reliable data on the single-cell level with comparatively little effort and was therefore used in this study. Additionally, the fluorescence distributions obtained from flow cytometry can be used for further data analyses, such as statistical evaluation of process data and mathematical modelling of virus protein production and virion release. The transformation of arbitrary fluorescence intensities into standardised molecule equivalents of soluble fluorochrome (MESF) increases the comparability of obtained result between different experiments and cytometers [26].

The use of flow cytometry for indirect detection of virus-infected cells with polyclonal antibodies against herpes simplex virus type 1 (HSV-1) and influenza C virus was presented by Steele-Mortimer et al. [27]. The method included detection of viral infection in trypsinised non-fixed MDCK cells combined with viability discrimination. Lonsdale et al. [28] have reported a flow cytometric detection of infection status of Per.C6 suspension cells fixed in paraformaldehyde (PFA) by use of fluorochrome-labelled murine monoclonal antibodies against viral nucleoprotein (NP). Until now however, no method has been reported which covers the full range of premises for effective monitoring of cellular influenza A virus infection in bioreactor samples as addressed above.

In the following, we present a sensitive and quantitative method for the flow cytometric detection of influenza A virus infection with monoclonal antibodies in adherent MDCK cells fixed in ethanol and PFA/ethanol. This method is based on a ready-to-use mixture of fluorochrome-labelled murine monoclonal antibodies against human influenza virus strain A (H1N1) NP and matrix protein 1 (M1). Both M1 and NP are synthesized in the cytoplasm of the host cells soon after infection and are transferred into the nucleus for ribonucleoprotein packaging [22]. The mixture of antibodies against M1 and NP used in this study was not only suitable for the detection of human influenza A (H1N1) virus proteins, but could also be used for labelling other influenza A virus subtypes and species. This was shown for equine (H3N8) and porcine (H1N2) influenza viruses, thus implying a broader application range of the antibody mixture as reported for the use of the antibody against NP only [23,28]. Data obtained from infections at a high multiplicity in bioreactors show the potential of this assay for monitoring virus production processes. This also implies that the method might be beneficial for complying with requirements of PAT guidelines.

## Results

### 1. Qualitative detection of different influenza A virus infections in MDCK cells

According to the recommendations of WHO, influenza virus strains used for vaccine manufacturing are adapted each year depending on the expected prevalent virus strains. For a broad application range, detection of influenza A virus strains covering a broad subtype spectrum is desirable. In addition, detection of strains derived from various hosts would be desirable. The antibodies used in this study originally targeted human influenza A virus (H1N1) M1 and NP. In order to verify if influenza virus strains from other hosts could also be detected with these antibodies, cells infected with equine influenza A virus (H3N8) were analyzed by flow cytometry and laser-scanning microscopy.

**Figure 1:**
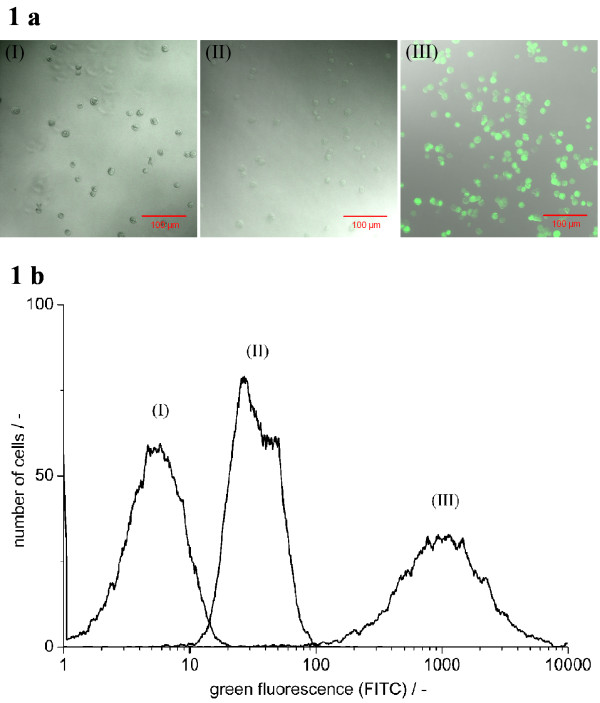
**Detection of equine influenza A virus infection in MDCK cells**. 1a: Fluorescence microscopy (LSM510, Zeiss: 400×, excitation: 488 nm, emission: 520–530 nm); 1b: flow cytometry. Uninfected cells: unstained (I) and stained against influenza A virus M1 and NP (II); infected cells: equine influenza A virus-infected, 18 h p.i., stained against influenza A virus M1 and NP (III).

Laser-scanning microscopy was used for qualitative assessment of the fluorescence-labelling of equine influenza virus-infected samples (fig. 1a). Uninfected cells were used as negative control. Microscopical images of uninfected unstained cells (fig. 1a (I)) and uninfected cells stained with antibodies against human influenza A virus M1 and NP (fig. 1a (II)) did not show a difference in fluorescence. As expected, cells infected with equine influenza virus (fig. 1a (III)) showed a strong increase in green fluorescence. The same samples were measured by flow cytometry (fig. 1b). With flow cytometry, the unstained uninfected cells (fig. 1b (I)) showed lower fluorescence than the stained uninfected cells (fig. 1b (II)) while equine influenza virus infected cells (positive control, (fig. 1b (III)) showed strong fluorescence. The fluorescence of the unstained uninfected cells (fig. 1b (I)) was due to cellular auto-fluorescence. The fluorescence increase of the uninfected stained cells (fig. 1b (II)) compared to the unstained cells (I) represented unspecific binding of the antibodies to cellular components. The increase of fluorescence between stained uninfected (fig. 1b (II)) and infected (fig. 1b (III)) cells, detectable by both microscopy and flow cytometry, was based on specific binding of at least one of the antibodies.

**Figure 2:**
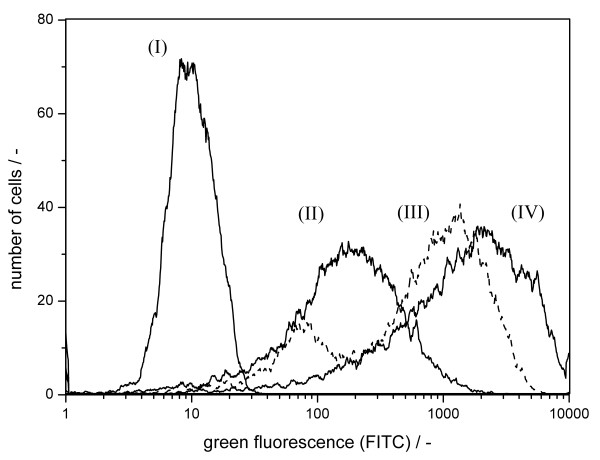
**Infection of MDCK cells by different influenza A virus strains**. Immunocytometric detection of cells stained with antibodies against influenza A virus M1 and NP. (I): uninfected cells; (II): cells infected with equine influenza A virus H3N8 (18 h p.i.); (III): cells infected with porcine influenza A virus H1N5 (18 h p.i.); (IV): cells infected with human influenza A virus H1N1 (18 h p.i.).

The detection of influenza A virus infected cells was also tested with cells infected by viruses of other host specificity and subtype. Therefore, cells infected by human influenza A virus (H1N1) and porcine influenza A virus (H1N2) were compared to equine influenza A virus (H3N8) infected cells (fig. 2). Cellular infection with all influenza virus strains caused a strong increase of the flow cytometric signal (fig. 2 (II), (III), (IV)) compared to the stained but uninfected cells (fig. 2 (I)). The results indicated specific binding of the antibodies not only to human and equine, but also to porcine influenza A virus proteins. Western blot analysis showed specific binding of the antibodies against M1 and NP to human influenza M1 and NP, respectively (data not shown). Specific binding of the anti-M1 antibody was also observed for equine influenza M1. However, the anti-NP antibody showed strongly decreased binding affinity to equine influenza NP. Thus, the detected fluorescence signals do not necessarily reflect binding of both antibodies in the mixture. In the following, equine influenza virus infection was used to show applicability of the method.

A crucial aspect, in particular for quantitative analysis, is elimination of artefacts during preparation of control samples for flow cytometry. Such artefacts might lead to erroneous discrimination settings for uninfected cells. In order to select the best negative control sample for quantitative analysis, cells harvested at the end of the cell growth phase were compared to mock-infected cells.

**Figure 3:**
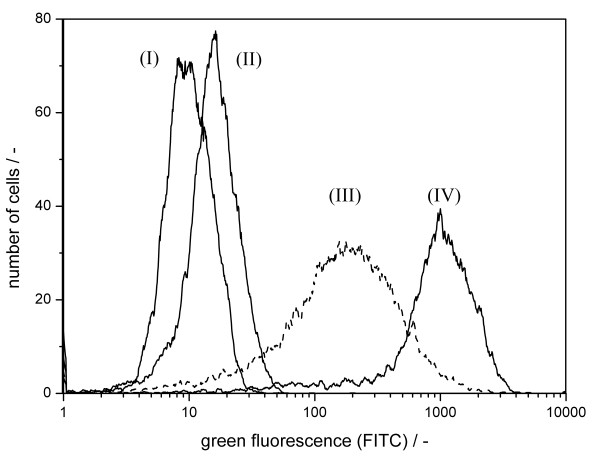
**Influence of time of harvest on negative controls and infected cells**. Immunocytometric detection MDCK cells stained with antibodies against influenza A virus M1 and NP: Negative controls: uninfected cells (I), mock-infected stained cells, harvested 2 h p.i. (II); infected cells: equine influenza A virus-infected, harvested 18 h p.i. (III), equine influenza A virus-infected, harvested 6 h p.i. (IV).

During vaccine production in MDCK cells, serum-containing cell growth medium is replaced by serum-free virus maintenance medium with intermediate washing steps prior to influenza virus infection. The influence of these changes in culture conditions on unspecific antibody binding in uninfected cells was investigated. Mock-infection simulated the procedure during the preparation for viral infection (without adding the virus seed), thus representing the treatment of infected cells more precisely than the use of cells harvested from growth medium. Two types of negative control were used: uninfected cells harvested from cultivation in cell growth medium (CGM) without medium exchange (see also fig. 2, (I)) and mock-infected cells (see above). Flow cytometric analysis revealed an increase in green fluorescence in mock-infected cultures (fig. 3, (II)) compared to uninfected cells (fig. 3 (I)). Hence, the washing procedure and medium change from CGM to virus maintenance medium (VMM) led to an increase in unspecific fluorescence independent of viral infection (table 1). Compared to mock-infection, the use of uninfected cells harvested from cell growth medium as negative control would have introduced an undesired underestimation of unspecific antibody binding leading to an overestimation in the quantification of infected cells. Therefore, mock-infected samples were used as negative controls in all further experiments.

**Table 1:** Quantification of flow cytometric measurements (n = 5; 1.0*10^4 ^cells per sample).

	uninfected	mock-infected	Infected
mean i_m_	13.0	18.2	261.7
mean c_MEF_	4.19 *10^4^	5.98 *10^4^	9.02 *10^5^
RSD of mean c_MEF_	± 8.45	± 2.46	± 1.66
**c_i_**		**0.0**	8.42 *10^5^

i_m _= fluorescence intensity measured by flow cytometry

c_MEF _= number of MEF per cell, according to calibration of fluorescence with Sphero

FITC Calibration particles (eq. 1, fig. 5)

c_i _= number of MEF per cell bound specifically to virus proteins in cells (eq. 2)

Another aspect examined was the dependence of the immunofluorescence on the time of harvest post infection. In order to asses this, MDCK cells were infected with equine influenza virus at a moi of 1.0 and harvested 6 h p.i. and 18 h p.i., respectively. These cells were measured flow cytometrically (fig 3). Compared to cells harvested 6 h p.i. (fig. 3 (IV)), cells harvested 18 h p.i. (fig. 3 (III)) showed a reduced mean fluorescence intensity. Furthermore, their fluorescence distribution was broader. The differences in measured fluorescence intensity indicated changes of intracellular influenza A virus M1 and NP content during the course of infection. The choice of the cell fixative depends on the cellular feature being investigated [29].

Fixation and storage in ethanol was reported to be suitable for long-term conservation [30], which is beneficial for the intended application. The influence of ethanol fixation on the stainability was therefore compared to PFA fixation with subsequent ethanol fixation to investigate which fixation procedure yields better results (data not shown). Ethanol fixation led to a decrease in unspecific immunostaining with mock-infected cells and an increase in specific immunofluorescence of equine influenza virus-infected cells compared to PFA/ethanol fixation. Thus, as the choice of the fixation method was not restricted by fixation requirements of additional staining, ethanol fixation was favoured over PFA/ethanol fixation. Both fixation methods were found to inactivate infectious samples, which facilitated the handling of virus material.

### 2. Quantitative detection of equine influenza A virus infection in MDCK cells

The fluorescence distributions measured by flow cytometry were used to quantify the status of infection (i.e., to distinguish between infected and uninfected cells). Cultivation conditions preceding infection (washing steps, medium exchange) also had an impact on unspecific cell fluorescence, as shown by differences in fluorescence of uninfected compared to mock-infected cells (see fig. 3, and table 1). Mock-infected cells reproduced unspecific fluorescence changes occurring in infected cell cultures better than uninfected cells and were therefore used as negative controls. Cells with fluorescence higher than 99% of the mock-infected cells were defined as infected. Correspondingly, the fluorescence threshold for the detection of an infected cell was set equal to the quantile (mean x_0.99_) of the cumulative fluorescence distribution (F) of mock infected cells. The fluorescence intensity at x_0.99 _did not only depend on the mean fluorescence intensity of the mock-infected cells (mean i_m_) but also on the corresponding fluorescence distribution width.

**Figure 4:**
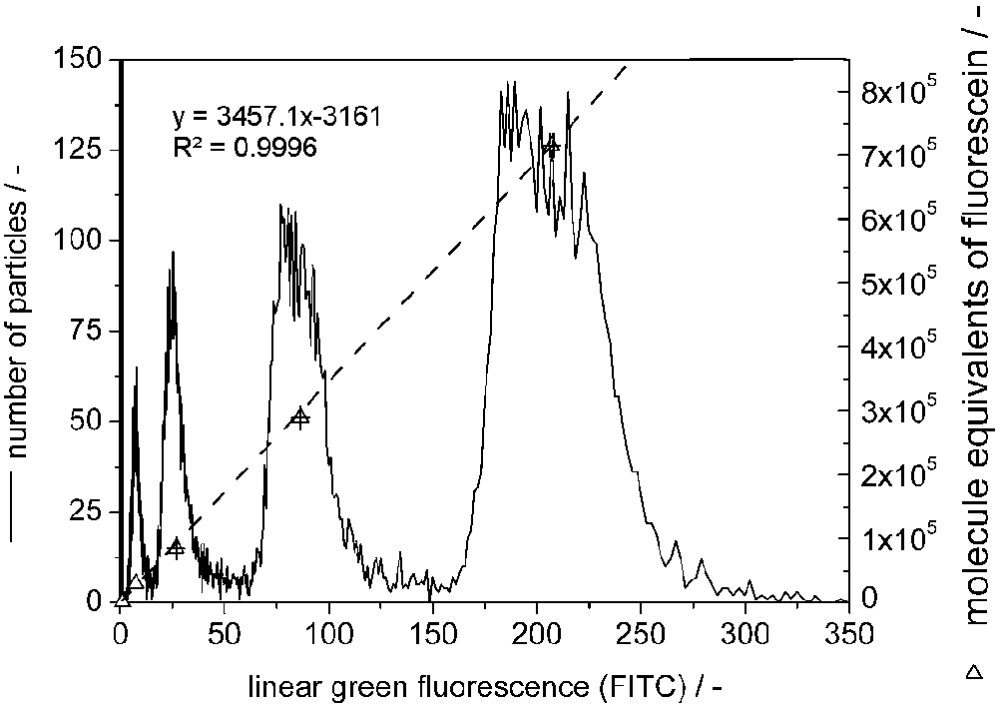
**Fluorescence intensity calibration**. Flow cytometric measurement of Sphero FITC Calibration particles (–: linear display after logarithmic data acquisition). For each particle population the mean fluorescence intensity was calculated from three samples (mean of i_m_: Δ ± S.D.), fitted by linear regression (---).

In order to compare measured fluorescence intensities of cell populations, a fluorescence calibration was done using Sphero FITC Calibration particles (fig 4). Accordingly, the fluorescence intensities could be transformed to numbers of molecules of equivalent fluorescein. This standardisation allows comparisons of signal intensities not only within samples stained in a group but also within different staining groups, e.g. measurements of different bioprocess batches. The difference in the molar quantum yield between fluorescein conjugated to calibration particles and to antibody molecules was not taken into account. Thus, the fluorescence from antibody binding measured by flow cytometry was expressed as molecules of equivalent fluorescein (MEF) as calibrated with fluorescent particles. The number of MEF per cell cMESF was calculated from the measured fluorescence intensity (i_m_) taking into account the slope of the calibration curve (eq. 1).

(1) *c*_*MEF *_= 3457.1 · *i*_*m *_- 3161

The fluorescence threshold of samples taken at time of infection (t = 0 h) was used to determine the number of MEF bound unspecifically to cells (c_MEF,*t *= 0_). The number of MEF bound specifically to virus proteins of an infected host cell (c_i_) was calculated by subtracting the number of unspecifically bound MEF (c_MEF,*t *= 0_) from the measured total number of MEF per cell (c_MEF,i_, eq. 2).

(2) *c*_*i *_= *c*_*MEF,i *_- *c*_*MEF,t *= 0_

Mock-infected cells were stained and analysed to detect the increase in background fluorescence at time of infection (toi) caused by washing steps and medium exchange (table 1). Equine influenza virus-infected cells (harvested 18 h p.i.) were stained and analysed. The number of MEF specifically bound per cell was calculated by subtracting the calculated mean MEF in mock-infected cells from the mean MEF in infected cells. The average number of MEF per cell was 8.42*10^5 ^in equine influenza virus-infected cells harvested 18 h post infection.

**Figure 5:**
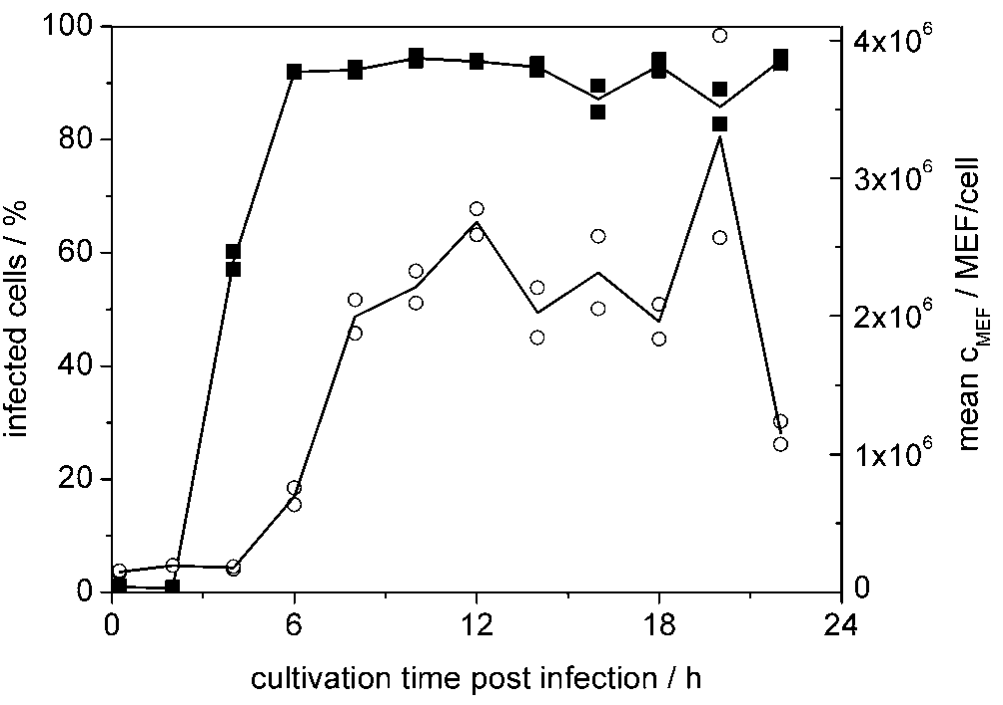
**Quantitative detection of equine influenza A virus infection**. Cellular infection status (■) and measured fluorescence intensity during infection of MDCK cells with equine influenza virus in a lab-scale bioreactor (1 l wv, moi = 3.0); duplicate measurements (symbols) and profile of the corresponding mean (lines).

To characterize the time course of the fraction of infected cells in a single-step infection experiment, MDCK cells were infected with equine influenza virus at a moi of 3.0 (fig. 5). The cells were cultivated on microcarriers in a lab-scale bioreactor (1 l working volume). Cells harvested at time of infection were used as negative controls for the determination of the fluorescence threshold. For the quantification of the fraction of infected cells at x_0.99 _a minimum signal intensity (c_MEF_) of 3.58*10^5 ^MEF was necessary. Between 2 to 4 h p.i. the mean cMEF of all measured cells increased, indicating the onset of intracellular accumulation of synthesized virus proteins. Most of the expected fraction of infected cells (close to 100% of all cells) could be detected at 6 h post infection. Thus, this was considered the earliest time-point of quantification of cellular infection. The percentage of infected cells remained relatively constant (mean: 90.0% ± 4.9%) up to 22 h p.i. and quite unaffected by changes of the mean fluorescence, especially between 16 to 22 h post infection. During this time the measured fluorescence intensities showed large variations (mean c_MEF_: 2.18*10^6 ^± 41.9%), probably indicating variations in virus protein content per cell over time of infection.

### 3. Vaccine production process monitoring: infection status of cells and virus titres

**Figure 6:**
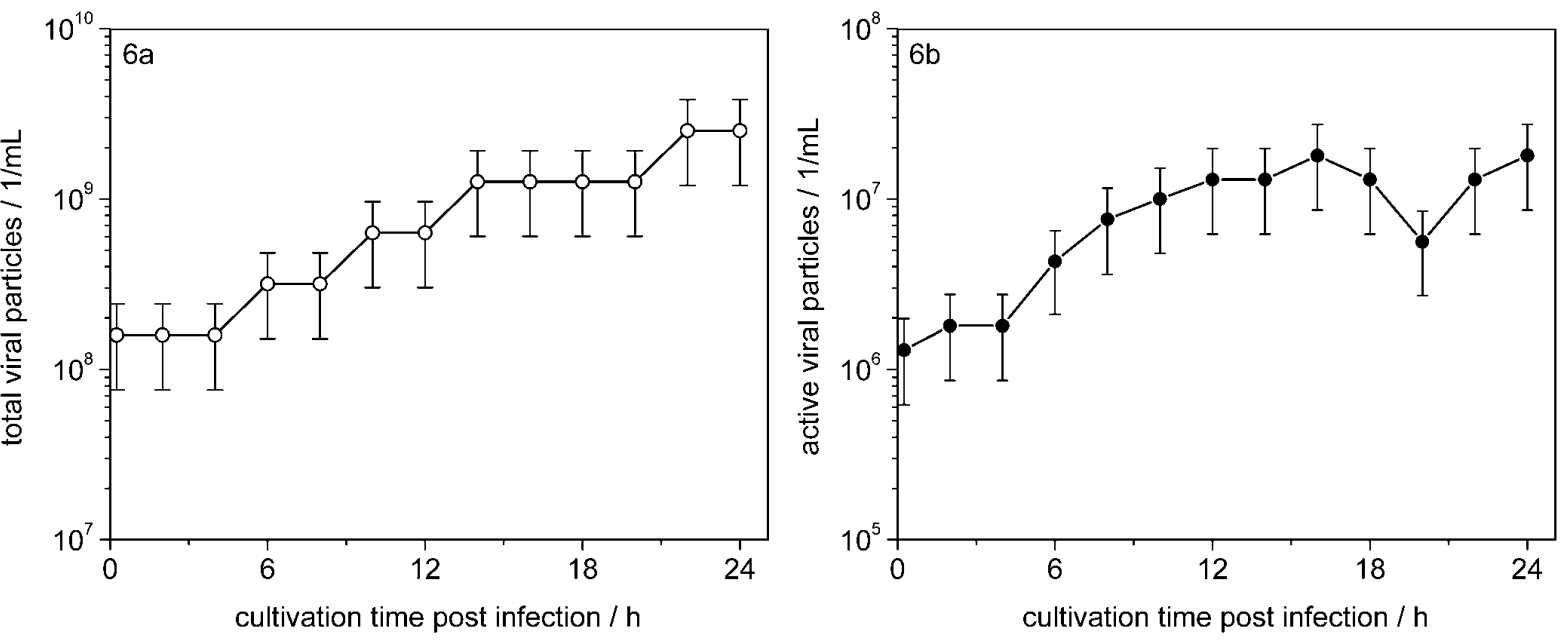
**Comparison of standard titration methods with flow cytometry for monitoring virus infection**. Equine influenza virus infection of MDCK cells in a lab-scale bioreactor (1 l wv, moi = 3.0, last sample not analyzed by flow cytometry due to cell lysis); **6a**: total virus particle concentration measured by hemagglutination assay (∘ ± standard error of the method); **6b**: infectious virus concentration determined via virus infectivity assay (TCID_50_, ∙ ± standard error of the method).

Commonly, virus titrations using hemagglutination, plaque formation or immunostaining are used to quantify the concentration of virus particles in cell culture supernatants during vaccine production. Infectious and non-infectious influenza virions are released by budding from the apical surface of their host cells, which is preceded by viral genome replication and intracellular virus protein accumulation. For a comparison of the flow cytometric detection of cellular infection status with standard titration methods, MDCK cells were cultivated on microcarriers in a lab-scale bioreactor (1 l wv). The cells were washed and infected with equine influenza A virus (H3N8) at a moi of 3. The time course of the cellular infection status is shown in fig. 5. Additionally to flow cytometry, the progress of virus infection was monitored by analysis of extracellular virus particle production. Total and infectious virus particle release into the culture supernatant was analysed with hemagglutination (fig. 6a) and TCID_50 _assay (fig. 6b), respectively. The comparatively high viral titres at the beginning of infection reflect the high moi used for this cultivation. The concentration of total virus particles increased over time from 0.9 log HA units/100 μl to a maximum of 2.1 log HA units/100 μl at 22 h post infection. Correspondingly, the concentration of infectious viruses increased from 1.3 *10^6 ^to 1.8 *10^7 ^virions/ml with an intermediate decrease to 5.6 *10^6 ^virions/ml between 16 and 22 hours.

The onset of intracellular virus production could be detected by an increase in the fluorescence (c_i_) at 4 h p.i. (fig. 5). At this time point about 60% of the cultured cells were clearly infected, whereas no increase in total virus particle number in the supernatant was detected via the HA assay (fig 6a).

Between 4 to 6 h p.i., the first virus particles were released from infected cells, as measured by the increase in HA and TCID_50_. At 6 h p.i., the percentage of infected cells was already close to its maximum, while a first maximum in the cellular fluorescence was reached at about 10 h p.i. (fig. 5). Obviously, the onset of virus particle release already took place prior to the maximum accumulation of viral antigen molecules in the host cells. Thus, the fluorescence intensity represents a net result of intracellular virus protein production and release of virus particles.

The increase in cellular fluorescence at 16 h p.i. and its decrease at 20 h p.i. was possibly caused by a population of cells infected during a second infection step at a later time-point, despite the comparatively high moi of 3 used in this study. This could also be concluded from the increase in infectious and total viral titres 20 h post infection.

## Discussion

We tested a ready-to-use fluorochrome-labelled monoclonal antibody mixture for the detection and monitoring of influenza A virus infection in fixed MDCK cells. The results from fluorescence microscopy and flow cytometry indicated that the combination of antibodies used is suitable not only human influenza A strains, but also for the detection of equine and porcine influenza A virus infection in cultured adherent MDCK cells. Furthermore, it was demonstrated that the assay is applicable to virus strains of different subtypes (human: H1N1, equine: H3N8 and porcine: H1N2). This indicated that the antibody mixture could be used for an even broader range of virus strains and host species origins than previously reported for the antibody against NP only [28].

The method was shown to be suitable for the measurement of fixed samples taken at different time-points during an experiment (e.g., bioreactor cultivations (fig. 5)). In contrast to the measurement of unfixed cells as reported by Steele-Mortimer et al. [27], it was possible to collect samples and stain them simultaneously to reduce sample preparation effort. Furthermore, both ethanol and PFA/ethanol fixation resulted in virus inactivation of the samples (data not shown). This effect led to increased biosafety and facilitated handling during sample preparation for flow cytometry.

The measured fluorescence from samples of equine influenza virus-infected cultures taken at different time-points suggests a time-dependent behaviour of the immunofluorescence signal (fig. 3). This is most likely due to the production and release kinetics of viral proteins of infected cells [22,28,31].

The earliest time-point of detection of virus protein accumulation was between 2 to 4 h p.i., which is consistent with the findings for the microscopic analysis using an antibody against influenza A virus NP only [23]. Compared to fluorescence microscopy, flow cytometry facilitates monitoring of thousands of cells, providing extensive data sets for mathematical analysis of virus dynamics with regard to spreading of the infection in cell populations. A calibration of arbitrary fluorescence intensities in terms of MEF increases the comparability of different experiments. Quantification of the fraction of infected cells was possible at about 6 h post infection, which is in good agreement with Lonsdale et al. [28], who have shown the quantification of human influenza A virus infection in PFA-fixed PER.C6 suspension cells at 5 h post infection.

In comparison with two titration methods routinely used in vaccine manufacturing, flow cytometry could detect viral infection at an earlier stage (fig. 5 and fig. 6). However, moi used in vaccine production is usually much lower to reach optimal product yields. Most likely, the advantage of flow cytometric monitoring would be even bigger as accumulation of virus proteins in individual cells could be detected long before significant amounts of virus particles are released for titration assays (the detection limit of the hemagglutination assay is about 2.0 *10^7 ^virions/ml).

After 6 h p.i. the fraction of infected cells, calculated from cytometric measurements, appeared to be stable, despite changes in the fluorescence intensity that occurred during the following infection phase (fig. 5). An underlying mechanism for these changes might be an initial intracellular accumulation of produced virus proteins followed by packaging and release of viral particles starting at a later stage of viral replication. This is supported by the fact that virus release, monitored by HA and the TCID_50 _assay, was not detected before 4 to 6 h p.i. (fig. 6a and 6b), whereas intracellular virus protein synthesis was detected already at 2 to 4 h p.i. by flow cytometry (fig. 5). Accordingly, the measured fluorescence intensities per cell appear to display the net result of intracellular virus protein accumulation and release via virus budding. An alternative explanation might be that the moi of 3 used to initiate this single-step infection experiment was not sufficient to infect all cells simultaneously. Consequently, a certain percentage of cells could have started to release virions with a delay resulting in a similar time course in HA and TCID_50 _titres. Additional experiments are currently being performed to elucidate this finding.

## Conclusion

In conclusion, flow cytometric monitoring of influenza A virus infection in adherent MDCK host cells using a ready-to-use mixture of antibodies against influenza A NP and M1 is applicable to a broader range of virus strains than previously reported. Due to its sensitivity and its robustness to process modifications – such as seed virus (host specificity and subtype), multiplicity of infection and cell concentrations – flow cytometry is a useful addition to standard titration methods for the monitoring of influenza A virus replication. Furthermore, it allows the generation of large data sets for statistical process analysis and validation of mathematical models for virus dynamics.

The application of this monitoring method in vaccine manufacturing may give new insights on virus infection dynamics supporting process development and optimization. For example, characterisation of the influence of moi on virus spreading and virus yields for different virus subtypes. In established processes, the method could be used for process analysis according to the PAT initiative to complement analysis of production runs, to facilitate interpretation of the impact of process variations on product quality or to support measures for improving batch to batch reproducibility.

## Methods

### Cell line

Madin-Darby canine kidney cells were obtained from ECACC (No. 84121903) and used for viral infection studies as described previously [7]. The adherent cells were expanded in cell growth medium (CGM): GMEM supplemented with 10% FCS (Gibco #10270-106) and 2 g/L peptone (International Diagnostics Group #MC33) in T-flasks and small-scale microcarrier cultures (Cytodex 1, GE Healthcare).

### Viruses

Equine influenza strain A/Equine 2 (H3N8)/Newmarket/1/93 (NIBSC), human influenza strain A (H1N1)/Puerto Rico/8/34 (NIBSC) and porcine influenza A/Swine (H1N2)/Bakum 1832/00 ([32,33]; seed virus was a kind donation from B. Hundt, Impfstoffwerke Dessau-Tornau) were used to infect MDCK cell cultures. After cell expansion in CGM the cultured cells were washed with phosphate-buffered saline (PBS) and further cultivated in FCS-free virus maintenance medium (VMM) containing 1 mg/l porcine trypsin (Gibco #27250-018) as described previously [7]. Mock-infected cultures were treated like infected cultures but without addition of seed virus.

### Preparation of standard samples

Uninfected MDCK cells from tissue flasks were harvested and fixed after four days of cultivation in CGM (negative control). Mock-infected MDCK cells were cultivated accordingly, harvested and fixed 1 h after the medium exchange (mock-infected negative control).

For positive controls, MDCK cells infected after 4 days (moi = 1.0) were used. Equine and human influenza A virus infected cells were harvested 18.0 h post infection (p.i.), whereas porcine influenza A virus infected cells were harvested 24.3 h post infection. All standard samples were stored in aliquots at -20°C until further analysis.

### Bioreactor cultivation

Small-scale bioreactor cultivations of MDCK cells were performed in a bioreactor (1 L wv) equipped with a glass ball agitator (DasGip). Cytodex 1 microcarriers were used at a concentration of 2 g/l (about 8 000 particles/ml). After a cell growth phase of 95 h the cells were washed with PBS and CGM was replaced by the same volume of VMM. Then the cells were infected with equine influenza virus (moi = 3.0).

### Hemagglutination assay

Titration of total viral particles was based on the method by Mahy and Kangro [10] with modifications described by Genzel et al. [7]. Titres are reported as log HA units per test volume (100 μl). Total virus particle concentrations were calculated from HA units, assuming that at the last dilution of virus showing complete agglutination, the ratio of red blood cells (RBC) and virus particles is equivalent (1) [9]. The concentration of red blood cells (c_RBC_) per ml test volume was kept constant (1*107 RBC/ml) for all measurements (eq.3).

(3) total virus particle concentration = c_RBC _* 10^(log HA titre/100 μl)^

The lower detection limit of the assay was 2.0*10^7 ^virus particles/ml. Due to a 2^n ^dilution of samples the standard error of the method was ± log 0.3.

### Virus infectivity assay

Infectious virus particle concentrations were determined as tissue-culture infectious dose (TCID_50_) titrations based on [10] with previously described modifications [34]. The quantification limit was 3.2*10^2 ^infectious virus particles/ml with a standard error of the method of ± log 0.3 due to 2^n ^dilution of samples.

### Cell harvest and sample storage

Cells from both tissue-culture flasks and microcarrier cultures were harvested for immunostaining of influenza virus infection.

Supernatants of tissue-culture flasks were centrifuged (60 g, 20 min, 4°C) to concentrate floating cells in a reduced volume. Adherent cells were washed with PBS and detached by trypsin/EDTA treatment (0.05% w/v trypsin, 0.02% w/v EDTA in PBS) at 37°C. Subsequently, detached cells and cells from supernatant were pooled. Samples from microcarrier cultivations were separated into two fractions by sedimentation of the microcarriers. The culture supernatants were centrifuged to concentrate floating cells as described for tissue-culture flask supernatants. The settled microcarriers were washed with PBS. In a subsequent step the adherent cells were detached from the microcarriers with trypsin/EDTA (0.5% w/v trypsin, 0.02% w/v EDTA in PBS). After cell detachment, the cells from culture supernatants were added again. Cells were separated from the empty microcarriers by sedimentation of the microcarriers.

From these pooled cell suspensions, 1.0*10^6 ^cells per aliquot were fixed in ethanol (70% v/v, -20°C) and stored at -20°C for at least 2 h. Infectious virus particles were inactivated by ethanol fixation (data not shown). For additional PFA fixation, the cells were kept in PFA (Sigma, #158127, 1% final concentration in PBS) for 20 min at room temperature. Afterwards, the samples were centrifuged (60 g, 20 min, 4°C) and washed once with PBS prior to ethanol fixation as described above.

### Immunostaining against influenza A virus M1 and NP

For the detection of influenza A virus infection, a mixture of murine monoclonal antibodies was used [35]. The ready-to-use antibody mixture (IMAGEN™ Influenza virus A and B, reagent A, DakoCytomation, # K6105) contained fluorescein-conjugated monoclonal antibodies (FITC-MAbs) against human influenza A virus NP and M1 at a concentration of 25 μg/ml each.

Ethanol-fixed aliquots of suspended cells were centrifuged at 100 g for 20 min at 4°C. The supernatant was discarded and the pellet was washed with 5 ml PBS containing glycine (2% w/v) and BSA (0.1% w/v). Subsequently, the samples were centrifuged at 100 g for 30 min at 4°C. The pellet was resuspended in 2 ml PBS with glycine and BSA, transferred into 2 ml reaction tubes and centrifuged at 60 g for 15 min at 4°C. After removal of the supernatant the pellet was dissolved in 25 μl of the FITC-MAb solution and incubated for 1 h in the dark at 37°C on a tube roller. Following the incubation, unbound antibody was removed by addition of 1.8 ml PBS and subsequent centrifugation (60 g, 15 min, 4°C). Finally, the pellet was resuspended in 0.5 ml PBS for flow cytometry or in 0.1 ml PBS for immunofluorescence microscopy, respectively. The samples were stored in the dark at room temperature until analysis.

### DNA content measurement for cell cycle analysis

In addition to immunostaining, the cell cycle distribution was determined via DNA content in bioreactor samples (data not shown). DNA content measurements were performed as previously described [36]. Briefly, ethanol-fixed cells were prepared for DNA content measurements according to [30] with the following modifications: centrifugation steps were carried out with 100 g for 20 min at 4°C to minimize cell loss, and propidium iodide (Sigma, # P4170) staining and RNA digestion was performed at 37°C for 40 min prior to subsequent flow cytometry.

### Calibration of molecules of equivalent fluorescein (MEF) from fluorescence intensity

Measured fluorescence intensities were standardised using a fluorescein-specific fluorescence calibration kit (Sphero FITC Calibration Kit, Spherotech, # ECFP-F1-5K). MEF values of the calibration beads were determined in comparison to fluorescein-conjugated beads (NIST) by the supplier. Accordingly, the MEF values were taken from the supplier's certificate of analysis. The calibration beads were diluted in PBS in order to avoid pH-based differences in fluorescence per fluorescein molecule between calibration and sample measurements [37]. The mean fluorescence intensity (MFI) was calculated from three measurements. The calibration curve resulting from linear regression of mean channel number versus MEF values of the corresponding beads resulted in a slope of MEF per fluorescence channel (see Results, chapter 2.: Quantitative detection of equine influenza A virus infection in MDCK cells). The labelling efficiency of the antibodies was 2.3 fluorescein molecules per antibody molecule as specified by the supplier.

### Flow cytometry

Cytometric measurements were performed with a Beckman Coulter Epics XL cytometer (Beckman Coulter) equipped with a 488 nm argon laser using the Expo32 software (Beckman Coulter). Two aliquots of each sample were stained and 1.0 * 10^4 ^single cells analyzed. Cells were distinguished from debris via forward-light scattering (FSC) and side-light scattering (SSC). Single cells were discriminated from cell aggregates using plots of forward-light scattering signal area (FSC-A) against forward-light scattering signal height (FSC-H). FITC fluorescence acquisition was performed with logarithmic binning.

Cells of bioreactor samples were stained with both propidium iodide and FITC-MAbs. Here, cell aggregates were distinguished from single cells via the signal ratio of red fluorescence signal area and signal peak.

For the discrimination between infected and uninfected cells mock-infected negative control samples were stained and analyzed. The fluorescence intensity border between infected and uninfected cells was set to a quantile (mean x_0.99_) of the cumulative distribution (F). Mean x_0.99 _was determined from two measurements of an immunostained negative control, using mock-infected or infected cells (harvested 0 h p.i.), respectively.

## List of abbreviations

FITC-MAbs: fluorescein-conjugated monoclonal antibodies; HA: hemagglutinin; M1: influenza A virus matrix protein 1; MDCK: Madin-Darby canine kidney cells; MEF: molecules of equivalent fluorescein; MFI: mean fluorescence intensity; moi: multiplicity of infection; NP: influenza A virus nucleoprotein; PAT: process analytical technology; PFA: paraformaldehyde; p.i.: post infection; PMT: photomultiplier tube; TCID_50_: tissue-culture infectious dose; toi: time of infection.

## Authors' contributions

JS designed and carried out the experimental study and drafted the manuscript. YG and UR originally conceived the study, supported interpretation of data and critically revised the manuscript. All authors read and approved the final version.
